# Right-lateralized fronto-parietal network and phasic alertness in healthy aging

**DOI:** 10.1038/s41598-020-61844-z

**Published:** 2020-03-16

**Authors:** Marleen Haupt, Adriana L. Ruiz-Rizzo, Christian Sorg, Kathrin Finke

**Affiliations:** 1 General and Experimental Psychology, Department of Psychology, Ludwig-Maximilians-Universität München, Munich, Germany; 2 Graduate School of Systemic Neurosciences (GSN), Ludwig-Maximilians-Universität München, Munich, Germany; 3 Department of Neuroradiology, Klinikum rechts der Isar, Technische Universität München, Munich, Germany; 4 Department of Psychiatry and Psychotherapy, Klinikum rechts der Isar, Technische Universität München, Munich, Germany; 5 Hans-Berger Department of Neurology, University Hospital Jena, Jena, Germany

**Keywords:** Cognitive ageing, Neural ageing

## Abstract

Phasic alerting cues temporarily increase the brain’s arousal state. In younger and older participants, visual processing speed in a whole report task, estimated based on the theory of visual attention, is higher in cue than no-cue conditions. The present study assessed whether older participants’ ability to profit from warning cues is related to intrinsic functional connectivity (iFC) in the cingulo-opercular and/or right fronto-parietal network. We acquired resting-state functional magnetic resonance imaging data from 31 older participants. By combining an independent component analysis and dual regression, we investigated iFC in both networks. A voxel-wise multiple regression in older participants yielded that higher phasic alerting effects on visual processing speed were significantly related to lower right fronto-parietal network iFC. This result supports a particular role of the right fronto-parietal network in maintaining phasic alerting capabilities in aging. We then compared healthy older participants to a previously reported sample of healthy younger participants to assess whether behaviour-iFC relationships are age group specific. The comparison revealed that the association between phasic alerting and cingulo-opercular network iFC is significantly lower in older than in younger adults.

## Introduction

Phasic alertness refers to momentary enhancements of the brain’s “state of readiness” due to warning cues^1^. Previous studies, using a whole report task with briefly presented letter arrays and parametric modelling of report performance based on the theory of visual attention (TVA)^2^, demonstrated that perceptual speed of visual processing is enhanced by visual^3^ and auditory warning cues^4^. Furthermore, auditory alerting was shown to be effective in both healthy younger and older participants^5^. Concerning neural correlates underlying phasic alerting effects in younger adults, we found that the individual cueing benefits were significantly related to intrinsic functional connectivity (iFC) in the cingulo-opercular network (CON)^6^. IFC refers to spatial patterns of correlated blood oxygen level dependent (BOLD) signal with a frequency of 0.01–0.1 Hz over time^7^. These BOLD fluctuations can be measured using resting-state functional magnetic resonance imaging (rs-fMRI).

With respect to older adults, it is unclear whether their preserved phasic alertness response relies on the same intrinsic brain network as in younger adults or a different one. Reviewing the importance of right-hemispheric structures for cognitive reserve^8^, Robertson suggested that the integrity and functional connectivity of the right fronto-parietal network (rFPN) is decisive for the late-life maintenance of attentional abilities and, especially, alertness functions^9,10^. He summarized evidence for a close, bidirectional relationship of the availability of noradrenaline provided by the locus coeruleus, which is decisive for the ability to increase arousal^11,12^, and the integrity of the right fronto-parietal network in aging individuals. The model is supported by a study demonstrating that  activating the noradrenergic system by applying a handgrip task increased functional connectivity between the locus coeruleus and the fronto-parietal network in older compared to younger adults^13^. Taken together, previous evidence and relevant cognitive reserve models suggest that connectivity in the rFPN could be essential for alertness functions in healthy older participants.

The present study, therefore, sought out to investigate whether iFC in the CON and/or rFPN do underlie phasic alerting effects in healthy aging. In order to address this question, we first examined the relationship between individual iFC in the CON as well as rFPN and the individual degree of the phasic alerting effects in a group of healthy older adults. Second, we investigated iFC-behaviour relationships in task-related sensory networks. The degree of the phasic alerting effects could potentially be related to auditory networks as they were induced by auditory cues. They could also be associated with visual networks as we measured the effect of these auditory cues on visual processing speed. All networks were identified by visual inspection and cross-correlation with networks reported in a prior relevant study^14^. As iFC in visual areas usually splits up into several visual networks and two visual networks yielded high cross-correlations, we decided to include both networks in our analyses (see Supplements for visualization of sensory networks). In addition to these intra-network analyses, we explored inter-network connectivity patterns between the CON, rFPN, auditory, and visual networks. In order to ensure the specificity of observed associations, we carried out intra-network and inter-network control analyses in other attention-relevant intrinsic brain networks, expecting that they would not be significantly related to phasic alerting effects. Lastly, we compared healthy older adults to a previously assessed sample of healthy younger participants^6^ in order to determine whether associations between phasic alerting effects and iFC are age group specific.

The analyses of rs-fMRI data follow the pipeline of a previous publication^6^. To facilitate the reading process, specifications concerning the acquisition, preprocessing, and analyses of rs-fMRI data are also added to the Methods section of the current manuscript. For all analyses, phasic alerting effects are quantified as differences in visual processing speed between a condition with an auditory cue compared to a no-cue condition. Visual processing speed is measured in a whole report paradigm based on the TVA^2^. For details regarding the TVA-based whole report procedure and estimation of visual processing speed, please refer to the previous publication of purely behavioural results^5^ and the Methods section.

## Results

### Phasic alerting effects in healthy older participants

The behavioural data of the original sample of 32 healthy older participants have already been analysed and reported elsewhere^5^. As one older participant was excluded from the present study due to extensive head motion, we report the behavioural results for 31 healthy older participants included in the present rs-fMRI study.

All participants completed a verbal whole report paradigm with half of the trials being preceded by an auditory cue. On the behavioural level, we are comparing estimates of visual processing speed in cue and no-cue conditions. Due to non-normal distributions of visual processing speed values, we applied both robust and Bayesian analyses. The main effect of cueing was significant (*Qa* = 6.333, p = 0.012). In Bayesian terms, it yielded anecdotal evidence that visual processing speed differed between the cue and no-cue condition (B_10_ = 1.016). We also analyzed whether two different cue target onset asynchronies (CTOA) would influence visual processing speed estimates. Neither the CTOA main effect (*Qb* = 1.899, p = 0.168, B_10_ = 0.406), nor its interaction with cueing (*Qab* = 0.046, p = 0.830, B_10_ = 0.247) were significant.

As phasic alerting effects did not differ in both CTOAs, we are using the absolute cueing effect averaged over both CTOAs as the behavioural variable of interest for the subsequent rs-fMRI analyses.

### Associations between phasic alerting effects and iFC in healthy older participants

#### Cingulo-opercular and right fronto-parietal network

**Figure 1:**
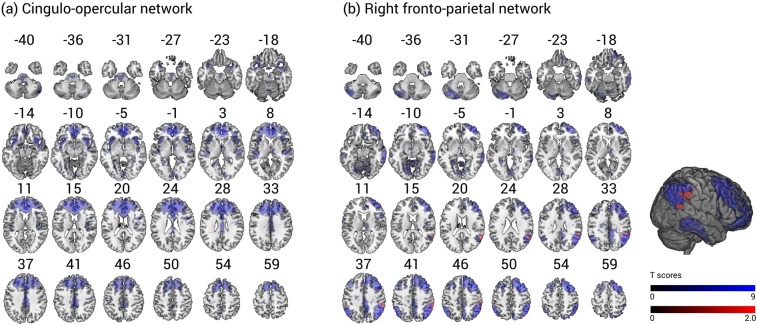
IFC in the cingulo-opercular network (**a**) and right fronto-parietal network (**b**) in older healthy participants. The clusters significantly related to phasic alerting effects (red) are overlaid on intra-network iFC (blue). The spatial maps are obtained by a combined independent component analysis dual regression approach. Behaviour-iFC associations were tested using a voxel-wise multiple regression, controlling for age, sex, head motion, and education (p < 0.05 FWE corrected at cluster level). The results are presented on a standard anatomical MNI152 template using MRIcroGL (https://www.mccauslandcenter.sc.edu/mricrogl/source); slice numbers in transverse plane are indicated.

We ran voxel-wise multiple regression analyses in the group of healthy older participants. First, we analyzed the behaviour-iFC associations in the rFPN, controlling for age, sex, education, and head motion. The analysis demonstrated that higher absolute cueing effects, averaged over both CTOAs, were significantly related to lower iFC in the rFPN, peaking in the right superior temporal gyrus (MNI coordinates in mm: [54 −52 22], cluster size: 647 voxels, T = 4.11, Z = 3.56, p = 0.022, FWE cluster-corrected) (see Fig. 1). Second, we analyzed behaviour-iFC relationships in the CON. We did not find significant associations of cueing effects and iFC in the CON.

Decisively, the significant peak of the behaviour-iFC relationship in the rFPN does not mean that the right superior temporal gyrus is specifically responsible or essential for phasic alertness. The interpretation of this finding is limited to the mentioned cluster best representing the behaviour-iFC association observed in the rFPN. This limitation applies because we used a voxel-wise analysis approach. By definition, iFC values of all voxels are relative values - they indicate the similarity of a voxel’s time course to the time courses of all other voxels in a certain network. Hence, we cannot interpret absolute values of voxels forming a significant cluster.

#### Auditory and visual networks

In order to address whether iFC in sensory networks is related to auditory phasic alerting effects on visual processing, we ran additional multiple regression analyses in the auditory and two visual networks (see Supplementary Figs. 1–3). The analyses yielded significant negative associations of phasic alerting effects and iFC in the auditory network, peaking in the left middle frontal gyrus (MNI coordinates in mm: [−28 12 48], cluster size: 521 voxels, T = 4.32, Z = 3.70, p = 0.042 FWE cluster-corrected), and visual networks. For visual network I (Allen component IC39) the peak was located in the left fusiform gyrus (MNI coordinates in mm: [−12 −82 −16], cluster size: 1434 voxels, T = 4.46, Z = 3.79, p < 0.001 FWE cluster-corrected); for visual network II (Allen component IC46) the peak was located in the left lingual gyrus (MNI coordinates in mm: [−30 −58 8], cluster size: 2135 voxels, T = 5.99, Z = 4.67, p < 0.001 FWE cluster-corrected).

#### Control analyses in other attention-relevant networks

To determine whether phasic alerting effects on visual processing speed are specifically linked to iFC in the right fronto-parietal network, we controlled for phasic alerting associations with iFC in other attention-relevant networks. The analyses yielded no significant association of phasic alerting effects on visual processing speed with iFC in the executive control and left fronto-parietal network (all p > 0.05 FWE cluster-corrected).

#### Inter-network connectivity analyses

Following the analyses of intra-network connectivity, we addressed whether, in addition, inter-network connectivity patterns of the CON or rFPN with other attention-related, auditory, and visual networks do underlie phasic alerting effects. The analysis yielded significant negative correlations of both visual networks with the CON. The left fronto-parietal, executive control, and auditory networks were positively correlated with the rFPN; both visual networks were negative correlated with the rFPN (see Supplementary Fig. 4). Importantly, none of these inter-network connectivity patterns were significantly related to the behaviour of interest, i.e. phasic alerting effects (−0.229 ≤ all r ≤ 0.259, all p ≥ 0.159).

In summary, the individual degrees of phasic alerting effects on visual processing speed in healthy older adults are primarily associated with their intra-network iFC in the rFPN, auditory, and visual networks. The results of the present study do not provide evidence for a relevant link between observed phasic alerting effects and inter-network connectivity patterns or intra-network iFC in any network but the rFPN.

### Age group comparison of associations between phasic alerting effects and iFC

Following the analyses in healthy older participants, we set out to compare the results of these participants to those of the previously analysed and reported sample of healthy younger adults who underwent the same behavioural and fMRI assessment procedures^6^. This comparison allows us to determine whether associations between phasic alerting effects and iFC in the CON or rFPN are age group specific.

**Figure 2:**
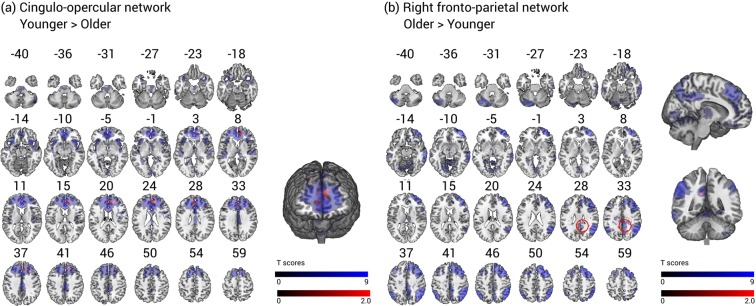
Age group differences of associations between phasic alerting effect on visual processing speed and iFC in the cingulo-opercular (**a**) and right fronto-parietal network (**b**). Panel a illustrates that the association between phasic alerting effects on visual processing speed and iFC in the cingulo-opercular network is significantly lower in older than in younger adults (p < 0.05 FWE corrected at cluster level, significant cluster in red). Panel b represents an exploratory analysis (p < 0.05 uncorrrected at cluster level) in the right fronto-parietal network. The spatial maps (blue) are obtained by a combined independent component analysis dual regression approach. Behaviour-iFC associations were tested using a voxel-wise multiple regression, controlling for age, sex, head motion, and education. The results are presented on a standard anatomical MNI152 template using MRIcroGL (https://www.mccauslandcenter.sc.edu/mricrogl/source); slice numbers in transverse plane are indicated.

First, we ran a voxel-wise multiple regression comparing behaviour-iFC relationships between healthy older and younger participants in the CON. This analysis revealed that the association between alerting benefits and iFC in the CON is significantly lower in older than in younger adults. The significant cluster is located in the superior orbito-frontal gyri bilaterally (peak MNI coordinates in mm: [14 40 16], cluster size: 1061 voxels, T = 4.20, Z = 3.90, p < 0.05 FWE cluster-corrected) (see Fig. 2a). While younger adults are characterized by a strong positive association between alerting benefits and iFC extracted from this cluster, older adults present a slight negative association (see Supplementary Fig. 5).

Second, we ran the same analysis in the rFPN. This analysis did not yield significant differences between age groups in their behaviour-iFC relationship (p < 0.05 FWE cluster-corrected).

Interestingly, the preceding analysis in healthy older participants provided strong evidence for a primary behaviour-iFC association in the rFPN. Therefore, we decided to perform an additional exploratory analysis without cluster correction but with a conservative voxel-wise threshold of p < 0.001. This exploratory multiple regression without cluster-level correction suggests that the association between phasic alerting effects and iFC in the rFPN is higher in older than younger participants (see Fig. 2b). The peak of the association was located in the right posterior cingulate cortex (MNI coordinates in mm: [10 −46 30], cluster size: 18 voxels, T = 3.87, Z = 3.62, p = 0.039 at cluster level, uncorrected). This result suggests that there is a trend for a stronger behaviour-iFC association in the rFPN in older versus younger participants.

## Discussion

Auditory warning cues increase visual processing speed in healthy younger and older participants^4,5^. In healthy younger adults, such phasic alerting effects have been linked to iFC in the CON^6^. The present study investigated whether the same or distinct functional connectivity patterns do underlie preserved phasic alerting effects in healthy older participants. Previous studies suggested that the rFPN could be essential for preserved alertness functions in healthy older participants^10,13,15^.

The present study sought out to identify iFC patterns underlying phasic alertness in healthy older participants. We addressed this question by measuring phasic alerting effects on visual processing speed in a TVA-based whole report and recording resting-state fMRI data. First, we analysed iFC-behaviour relationships in the CON and rFPN in healthy older adults. Second, we compared these iFC-behaviour relationships between healthy younger and older adults in order to determine whether associations in the CON and/or rFPN are age group specific.

In healthy older participants, higher absolute cueing effects were significantly related to lower iFC in the rFPN. In contrast to the results in younger participants^6^, we did not find significant behaviour-iFC associations in the CON in healthy older participants. Importantly, greater connectivity does not automatically imply better behavioural performance. Previous studies have demonstrated that lower behavioural scores were associated with higher iFC in the brain networks of interest^16,17^. Based on such evidence, Ferreira and Busatto^18^ summarised that the assumption “the more, the better” is overly simplistic and might be misleading in the interpretation of findings.

We also addressed iFC in the auditory network as we used auditory cues and in visual networks because we assessed the effect of alerting cues on visual processing. The analyses yielded significant negative associations of phasic alerting effects and iFC in the auditory network as well as both visual networks. Inter-network connectivity analyses of both the rFPN and CON with these task-relevant networks did not reveal significant relationships related to phasic alertness. However, as we have found significant behaviour-iFC associations in the auditory as well as two visual networks, we suggest that the rFPN in healthy older participants is in permanent, bi-directional exchange of information with sensory networks. On the one hand, the information it receives might be dependent on the efficiency of the auditory system to perceive auditory warning signals. On the other hand, the rFPN might accelerate the uptake of visual information by heightening the readiness of visual networks. The fact that we did not find significant behaviour-related connectivity patterns between networks, arguably, suggests that our connectivity measure lacks the sensitivity to reflect uni- or bi-directional communication between these brain areas. In this case, effective connectivity measures could facilitate the measurement of relevant directed connectivity patterns between the rFPN and the sensory network^19–21^. In addition, neither the intra-network connectivity of other attention-related networks nor their inter-network connectivity with the CON or rFPN were found to be significantly related to phasic alerting effects. These control analysis support the notion of the rFPN playing a specific role for phasic alerting effects in older participants.

Overall, the results of the behaviour-iFC analyses in healthy older participants indicate that right-hemispheric fronto-parietal structures are important for noradrenaline-mediated attentional and, particularly, alertness processes in aging^10,13,14^. Robertson^10^ summarized evidence suggesting that the integrity and functional connectivity in this network may facilitate attentional functions in old age. Enhancing visual processing speed in response to a warning signal depicts a central cognitive ability that can temporarily reduce the detrimental impact of age-related declines of general processing resources. Accordingly, older individuals with a high capability to mobilize additional capacity when faced with external warning signals are able to improve their rate of visual information uptake in critical situations. Older adults with lower phasic alertness capabilities might be characterized by a relatively stable capacity that is i) reduced due to aging effects and ii) does not flexibly adopt to task demands, even when provided with alerting environmental cues. A recent study, using a TVA-based paradigm with auditory alerting cues and measuring cue-related power and phase-locking effects in electroencephalography (EEG), documented that the variability of phasic alertness effects in older adults is reflected in the neural response. Namely, it was found that only those older participants with a relatively youth-like pattern of phase-locking showed reliable performance benefits^22^. The results of the present study suggest that these inter-individual differences in the ability to utilize auditory phasic alerting cues to increase visual processing speed are linked to iFC in the rFPN. They, thereby, support the importance of the integrity and connectivity of this network for active perception mechanisms and cognitive reserve in old age.

The comparison of healthy older participants with a previously reported sample of younger adults revealed that associations between phasic alerting effects and iFC in the CON are age group specific. The association between phasic alerting benefits on visual processing speed and iFC in the CON is significantly lower in older than in younger adults. This finding can be consolidated with a recent resting-state like fMRI study demonstrating reduced functional connectivity between the locus coeruleus, depicting the brain’s primary source of noradrenaline, and core salience network structures in healthy older compared to younger adults^23^. The age group comparison in the rFPN showed that behaviour-iFC pattern does not significantly differ between healthy younger and older participants. A potentially more important role of the rFPN in healthy aging is, however, suggested by an additional exploratory analysis without cluster correction. Based on the specific role of the CON in younger adults^6^ and the primary role of the rFPN in healthy older adults suggested by both current results and former studies^9,10,13^, we expected to observe an age group specific dissociation of iFC patterns underlying alerting effects in both networks. It is possible that our sample of healthy older participants is heterogeneous with regard to the relevance of iFC in the rFPN for phasic alerting effects. A recent study suggested that there are substantial individual differences in neural responses underlying phasic alerting effets in aging individuals^22^. Some aging individuals might preserve a youth-like functioning while others might be characterized by obvious changes. Such variance could also affect associations between phasic alerting effects and iFC in the CON and rFPN. The interpretation of results is, further, complicated by the question whether the relevance of the rFPN in aging individuals indicates a (more or less intact) compensatory mechanism relying on this system. Overall, future studies are needed to establish whether the association between alertness and iFC in the rFPN is age group specific.

The present study has several limitations. First, the cross-sectional design and regression analyses do not allow for inferences being drawn regarding directionalities between phasic alertness, aging, and iFC in the CON and rFPN. Second, our study lacks a direct link to the locus coeruleus-noradrenaline system as we did not include a specific readout. Neuromelanin-sensitive magnetic resonance imaging sequences are a promising tool for quantifying locus coeruleus intergrity^24,25^. In addition, measuring pupil dilation would provide a window into locus coeruleus activity (for a review see^15^). Third, our voxel-wise analysis approach does not allow for a direct comparison of behaviour-iFC relationships between networks as such analyses do not yield one value describing iFC on the network-level.

## Methods

### Participants

Thirty-two older adults (≥60 years) participated in the present study. One older participant had to be excluded due to extensive head motion. For the age group comparison, they were contrasted with 32 healthy younger (18–35 years) participants whose rs-fMRI data have been reported in a previous study^6^. The behavioural data of both younger and older participants have already been reported elsewhere^5^. The final sample of the present study consisted of 32 younger and 31 older participants (see Table 1). All participants reported normal or corrected-to-normal vision, and they were reimbursed for their participation. The study was reviewed and approved by the ethics committees of the Department of Psychology of the Ludwig-Maximilians-Universität München and the Klinikum rechts der Isar of the Technical University Munich. All methods were performed in accordance with the ethics protocol’s relevant guidelines and regulations. Written informed consent was obtained from all study participants in agreement with the ethics protocols.

**Table 1:** Demographics and visual processing speed (C) estimates of all participants.

Variable	Younger participants	Older participants
	N = 32	N = 31
mean age (SD)	26.6 (4.7)	71.1 (4.8)
sex (female/male)	20/12	10/21
handedness (right/left)	27/5	29/2
mean education in years (SD)	12.6 (0.9)	11.8 (1.8)
MWTB verbal intelligence score (SD)	28.6 (4.6)	32.7 (2.3)
MMSE score (SD)	—	28.9 (1.1)
Days between sessions^1^	240.1 (294.7)	255.0 (124.3)
Absolute Cueing Effect (over both CTOAs)^2^	—	1.65 (3.65)
Absolute Cueing Effect (long CTOA)^3^	3.92 (5.91)	1.69 (6.75)

*Note*. SD: standard deviation; handedness: assessed by Edinburgh Handedness Inventory; MWTB: Mehrfachwahl-Wortschatz-Intelligenztest, maximum score = 37 points; MMSE, Mini-Mental State Examination, maximum score = 30 points with values < 24 points indicating cognitive impairment; ^1^Number of days between behavioural and rs-fMRI session; ^2^((C_cue_ [long] − C_no-cue_ [long]) + (C_cue_ [short] − _Cno-cue_ [short]))/2; ^3^C_cue_ [long] − C_no-cue_ [long].

As in the younger sample, the acquisition of the rs-fMRI (approx. 1 hour) and the TVA-based behavioural assessment (1–1.5 hours) of the older sample took place on two different days. Participants also completed the Edinburgh Handedness Inventory^26^, a multiple choice German vocabulary test measuring crystallized intelligence called “Mehrfachwahl-Wortschatz-Intelligenztest” (MWTB)^27^, and the Mini-Mental State Examination (MMSE) as a screening for cognitive impairments indicative of beginning dementia^28^. None of the participants had to be excluded based on a cut-off criterion for cognitive impairment, i.e. a score below 27/30 points. Demographic information of both study groups is presented in Table 1.

### TVA-based whole report paradigm with alerting cues

The details of the applied TVA-based whole report procedure have already been reported elsewhere^5^. In short, TVA is closely related to the biased competition account^29^ and implies parallel processing of several visual objects competing for selection into a capacity-limited visual short term memory (vSTM) store. The probability that an object gets selected before the store is filled is proportional to its processing rate^2^. An increase of phasic alertness leads to a proportional increase in the processing rate of the object^30^. The sum of the processing rates of all objects present in the visual display is defined as the observer’s overall visual processing speed *C* (in elements per second)^31^. By definition, all items in a whole report paradigm share the same expectancy and subjective importance. Hence, an increase in the observer’s alertness induced by auditory warning cues will lead to a proportional increase in parameter *C*^30^. By comparing visual processing speed in conditions with and without warning cues, the individual phasic alerting effect can be estimated.

**Figure 3:**
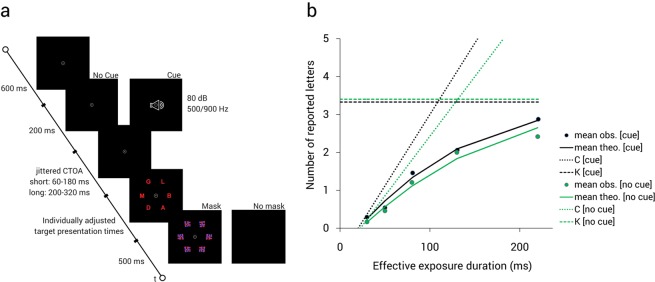
Exemplary trial sequence of the presented TVA-based whole report task (**a**) and summary of performance in cue and no-cue conditions for a representative healthy older participant (**b**). Solid curves (mean theo.) depict the best TVA-based fit to acquired data points (mean obs). The dashed line represents the model-based estimate of vSTM storage capacity *K*; the dotted line represents the model-based estimate of visual processing speed *C*.

All possible trial sequences can be seen in Fig. 3a. Participants were instructed to maintain central fixation throughout the task and verbally report all letters recognized with “fair certainty”, without any importance of speed or order. After entering all reported letters on the keyboard, the experimenter started the next trial with a button press. A scale presenting the individual’s accuracy rating based on all reported letters succeeded every test block. Participants were asked to maintain an accuracy level between 70% and 90%, with a deviating score leading to adapted instructions for the next test block. If the participants’ accuracy rating exceeded 90%, they were asked to also name letters that they believed to have recognized without complete certainty. If participants were less than 70% accurate, they were instructed to only report letters recognized with high certainty even if that meant that they would report fewer letters overall.

The whole behavioural experiment consisted of 8 blocks with 84 trials each. Half of the trials were preceded by an auditory cue and the other half of trials were uncued^5^. For the cue as well as no-cue conditions, 2 different CTOA spectrums, “long” and “short”, were used. The “short” CTOA had an average of 120 ms and was jittered around the average value in steps of ±20 ms, 40 ms, and 60 ms resulting in an overall range of 60–180 ms. The “long” CTOAs had an average of 260 ms with the same jittering steps leading to a range of 200–320 ms. For each trial, one CTOA was randomly drawn from the according CTOA distribution (short or long). In all conditions, the jittering was balanced with all jittered CTOAs having the same probability and appearing equally often across trials. The effects induced by the auditory alerting cue were comparable across the short and long CTOA spectrums in older participants^5^. Therefore, we used the average absolute cueing effect calculated as ((C_cue_ [long] − C_no-cue_ [long]) + (C_cue_ [short] − _Cno-cue_ [short]))/2 as the behavioural variable of interest in the subsequent iFC analyses in healthy older participants. Importantly, high-performing younger individuals demonstrated ceiling effects of visual processing speed in the short CTOA, derogating phasic alerting effects. As both age groups demonstrated stable phasic alerting effects in the long CTOA, we only analyzed the absolute cueing effect derived from long CTOA trials in the age group comparison (see Table 1).

The whole report task allows for the estimation of visual processing speed *C*, vSTM storage capacity *K*, and visual perceptual threshold *t0*. For detailed underlying estimation algorithms please refer to Kyllingsbaek^32^. Figure 3b shows the mathematically modeled exponential growth function of a representative older participant which is relating report accuracy (mean number of reported items) to effective exposure durations. The present study addresses the specific relationship between visual processing speed *C* and iFC as alertness cues predominantly affect visual processing speed^3–5^.

### Resting-state fMRI

The analyses of rs-fMRI data in the present study follow the pipeline described in a previous publication^6^. In order to facilitate the reading process, we repeat the specifications regarding data acquisition, preprocessing, Independent Component Analysis, Dual Regression, and statistical analyses in the following paragraphs. Previously reported rs-fMRI data of the younger participants^6^ are added to the present study to address potential differences between older and younger participants.

### Imaging Data Acquisition

Imaging data were acquired on a 3 T MR scanner (Philips Ingenia, Netherlands) using a 32-channel SENSE head coil. Small cushions stabilized participants’ heads in the head coil to reduce head motion. Earplugs and headphones reduced scanner noise. Functional data acquisition lasted for 12.5 minutes, and participants were instructed to keep their eyes closed, intend to stay awake, and to refrain from performing any cognitive or motor activity, i.e. be at rest, throughout the whole sequence. At the end of the sequence, all participants reported that they had stayed awake. The functional data set consisting of 600 volumes was acquired by multi-band echo-planar imaging (EPI)^33^ with a multi-band SENSE acceleration factor of 2 (TR = 1250 ms; TE = 30 ms; phase encoding in anterior-posterior direction; flip angle = 70°; field of view (FOV) = 192mm^2^; matrix size = 64 × 64, 40 slices with 3 mm thickness and an inter-slice gap of 0.3 mm; reconstructed voxel size = 3 mm × 3 mm × 3.29 mm). Structural data were obtained by a T1-weighted magnetization-prepared rapid-acquisition gradient echo (MPRAGE) sequence (TR = 9 ms; TE = 4 ms; flip angle = 8°; FOV = 240mm^2^; matrix = 240 × 240, 170 sagital slices; reconstructed isotropic voxel size = 1 mm).

### Imaging data preprocessing

The rs-fMRI data were preprocessed in MATLAB (R2017b, version 9.3.0.713579; The Mathworks Inc.) using SPM 12 version 6225 (https://www.fil.ion.ucl.ac.uk/spm/software/spm12/) and the Data Processing Assistant for Resting-State fMRI toolbox version 2.3 (DPARSF)^34^. After removing the first five functional volumes of every data set to account for T1 saturation effects, slice timing and head motion correction were performed by calling SPM functions. One older participant had to be excluded due to excessive head motion by the criterion of cumulative translation or rotation of 3 mm or 3° or due to more than 20% frame-wise displacements >0.5 mm (382/594 displaced frames)^35^. All images were manually reoriented to the AC-PC axis. The functional images were normalized into Montreal Neurological Institute (MNI) space with a 2-mm isotropic voxel size by unified segmentation to the structural image^36^. DPARSF integrates the three underlying procedures - coregistration, segmentation (grey matter, white matter, cerebrospinal fluid) and writing normalization parameters - into one processing step. The normalized images were smoothed using a 4 mm full-width-at-half-maximum (FWHM) Gaussian kernel. Additionally, band-pass filtering (0.01–0.1 Hz) was performed and the effects of nuisance covariates (whole-brain, white matter, and cerebrospinal fluid signals, as well as 12 head motion parameters, their derivatives, and scrubbing regressors) were removed.

### Independent component analysis and dual regression analyses

After preprocessing the functional data, we conducted a probabilistic Independent Component Analysis (ICA) in FSL (version 5.0.9), using the MELODIC command-line program version 3.14^37,38^. We specified 30 independent components aiming at decomposing the data of the rather heterogeneous sample of healthy younger and older adults into larger networks. We refrained from using more components in order to avoid a split-up of the data into smaller subnetworks. The ICA decomposed each time × space matrix into pairs of time courses and spatial maps on the group level. Subsequently, these files were used as input and a dual regression was employed in order to estimate spatial maps and time courses for each participant^39,40^. The dual regression approach allows to quantify the functional connectivity of each voxel with each spatial map while controlling for all other spatial maps within each participant^41^. Most importantly, we chose this approach as dual regression analysis is excelling in detecting inter-individual variability in functional connectivity compared to seed-based functional connectivity analysis^41^. In a first step, the group-average spatial map was regressed into the individual participants’ time × space matrices, resulting in 30 participant-specific time series. In a second step, the group-average time series was regressed into the same matrices, yielding 30 participant-specific spatial maps, i.e. one per independent spatial map on the group-level. The individual spatial maps contained Z-scores of every voxel within the according map. These Z-scores indicated the similarity of a particular voxel’s time course to the time course of the respective component on the group-level while controlling for all other components. Therefore, the voxel-wise Z-scores were used as input for statistical tests to analyse whether the given component derived Z-scores do relate to behavioural variables. Importantly, the results of the statistical analyses are solely related to the specific output of the ICA, i.e. independent components. These components represent intrinsic brain networks but the precise brain regions included may vary^41^. In a last step, the randomise permutation-testing tool (5,000 permutations, FWE-corrected p = 0.05) within the FSL framework yielded one-sample *t*-test or ‘group’ spatial maps^39,40^.

In order to identify typical intrinsic brain networks with our ICA-dual regression approach, we performed a spatial cross-correlation of our 30 independent components with intrinsic brain network templates derived from Allen *et al*.^15^, using the *fslcc* command of FSL. Accordingly, we identified the component with the strongest correlation coefficient with the “salience network” (component IC55, r = 0.35) and right fronto-parietal network (component IC60, r = 0.54) of Allen *et al*.^15^ as the CON and rFPN in the present study. These cross-correlations as well as a visual inspection of the brain areas included in the networks ensured that we identified the networks of interest for the present study. The CON comprises the cerebellum, amygdala, insula, basal ganglia, thalamus, paracingulate gyrus, anterior cingulate cortex, orbital gyrus, and frontal gyri (see Fig. 1a). The rFPN encompasses the cerebellum, prefrontal cortex, frontal gyri, intra-parietal sulcus, inferior parietal lobule, posterior cingulate cortex, and temporal gyri (middle and superior) (see Fig. 1b).

### Statistical analyses

#### Phasic alerting effects in healthy older participants

Due to non-normal distributions of visual processing speed violating the assumptions of general linear models, we applied an equivalent robust model^42,43^. We used a robust method based on 20% trimmed means for a 2 × 2 repeated-measures design with the within subject factors cueing (cue vs. no-cue) and CTOA (short vs. long) for visual processing speed. These analyses were performed using the WRS package^44^ in RStudio version 1.0.136^45^.

Apart from orthodox statistics, we also ran the Bayesian counterpart of repeated-measures ANOVAs^46^, using JASP version 0.8.5.1^47^. JASP calculates the Bayes factor which is a measure for the ratio of the likelihoods of two theories. By comparing those likelihood, the Bayes factor allows for a quantification of the evidence for each theory (e.g. null hypothesis and alternative, experimental hypothesis). Hence, if B_10_ is greater than 3 the present data substantially support the alternative hypothesis while values smaller than 1/3 are substantially favour the null hypothesis. B_10_ values between 1 and 3 (as well as 1 and 1/3 accordingly) solely yield anecdotal evidence for an hypothesis^48,49^.

#### Associations between phasic alerting effects and iFC in healthy older participants

##### Cingulo-opercular and right fronto-parietal network

The individual spatial maps resulting from the described second step of the dual regression served as input for the intra-network analyses conducted in SPM12 (http://www.fil.ion.ucl.ac.uk/spm/software/spm12/). We performed two voxel-wise multiple regressions of the absolute cueing effect averaged over both CTOAs on iFC in the CON and rFPN. For the analyses of behaviour-iFC associations in a given network, we performed significance testing for significance threshold p < 0.05 together with family-wise error correction for multiple comparisons at the cluster level (FWE cluster-corrected). We added age, sex, education, and head motion as planned covariates. We controlled for head motion by adding mean volume-to-volume head motion, i.e. frame-wise displacement, as a covariate to the multiple regression. We chose the measure by Jenkinson *et al*.^50^ as it considers voxel-wise differences in its derivation^51^.

##### Auditory and visual networks

We also identified task-relevant, sensory networks by visual inspection and cross-correlation. Subsequently, we tested for a significant relation between alerting effects and iFC in two visual networks (IC39, r = 0.37; IC46, r = 0.43) and an auditory network (IC17, r = 0.34) as our behavioural task consisted of visual stimuli and contained an auditory cue.

##### Control analyses in other attention-relevant networks

In order to address the specificity of the relationship between phasic alerting effects and iFC, we additionally performed control analyses in other attention-relevant networks. We identified them by visual inspection and cross-correlation with templates by Allen *et al*. (2011) as we did with the two networks of interest. We chose to control for alerting associations with iFC in the executive control network (IC71, r = 0.47) and the left fronto-parietal network (IC52, r = 0.42) as both networks have been reported to be associated with attentional processes in fMRI task studies^1,52^. The executive control network is distinguishable from the left and right fronto-parietal networks as it contains bilateral temporal gyri, bilateral precuneus, and the right precentral gyrus^15^. It does neither include frontal structures nor is it restricted to one hemisphere.

##### Inter-network connectivity analyses in healthy older participants

Furthermore, we explored whether phasic alerting effects are significantly associated with inter-network functional connectivity pattern between the CON or rFPN and task-relevant (auditory and visual) networks. We also controlled for inter-network connectivity between the two networks of interest and other attention-relevant networks. We addressed these questions by entering the individual time courses of the mentioned intrinsic brain networks (yielded by the first step of dual regression) into an inter-network analysis (using custom code written in MATLAB; also see^53^). We correlated the time course of the CON and rFPN with the ones derived from the other five attention- and task-relevant networks of interest per participant. Subsequently, we performed Fisher r-to-Z transformation and correlation analyses to test whether the inter-network connectivity patterns were significantly correlated with the absolute cueing effect averaged over both CTOAs.

##### Age group comparison

Finally, to address whether previously analysed associations between phasic alerting effects and iFC in healthy older participants are, indeed, age group specific, we entered healthy older as well as younger participants into intra-network analyses in the CON and rFPN. For both networks, we performed voxel-wise multiple regressions of the absolute cueing effect in the long CTOA split by age group on iFC values (p < 0.05 FWE corrected for multiple comparisons at the cluster level). We compared a vector including values for the absolute cueing effect for younger participants and zeros for older participants with a vector containing absolute cueing effect values for older participants and zeros for younger participants. As we were interested in the interactive effect of phasic alerting and age group on iFC, we controlled for the main effects of both age group and absolute cueing effect. Additionally, we added sex, education, and head motion as planned covariates.

## Supplementary information

Supplementary Material.

## Data Availability

The data and code used in this study are available upon request and data sharing complies with the institutional ethics approval.
