# Supplementary material for: Right-lateralized fronto-parietal network and phasic alertness in healthy aging
Source: Sci Rep. 2020 Mar 16;10:4823. doi: 10.1038/s41598-020-61844-z (PMC7075959; doi:10.1038/s41598-020-61844-z)
Supplement: Supplementary file 1 — Supplementary Material. [file 41598_2020_61844_MOESM1_ESM.pdf]

Supplementary Material

Right-lateralized fronto-parietal network and phasic alertness in healthy aging

Marleen Haupt, Adriana L. Ruiz-Rizzo, Christian Sorg, and Kathrin Finke

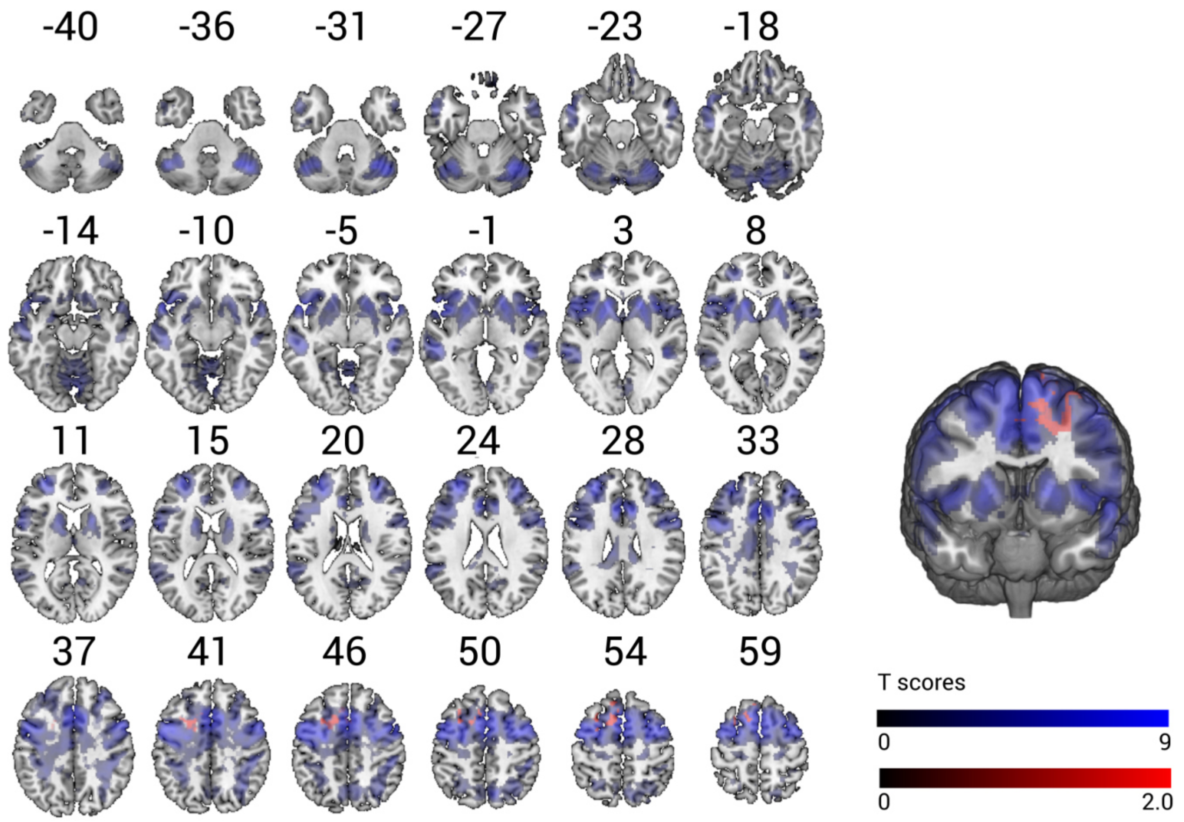

Figure S1. IFC in the auditory network in older healthy participants. The clusters significantly related to phasic alerting effects (red) are overlaid on intra-network iFC (blue). The spatial maps are obtained by a combined independent component analysis dual regression approach. Behaviour-iFC associations were tested using a voxel-wise multiple regression, controlling for age, sex, head motion, and education ($p < .05$ FWE corrected at cluster level). The results are presented on a standard anatomical MNI152 template using MRIcroGL (https://www.mccauslandcenter.sc.edu/mricrogl/source); slice numbers in transverse plane are indicated.

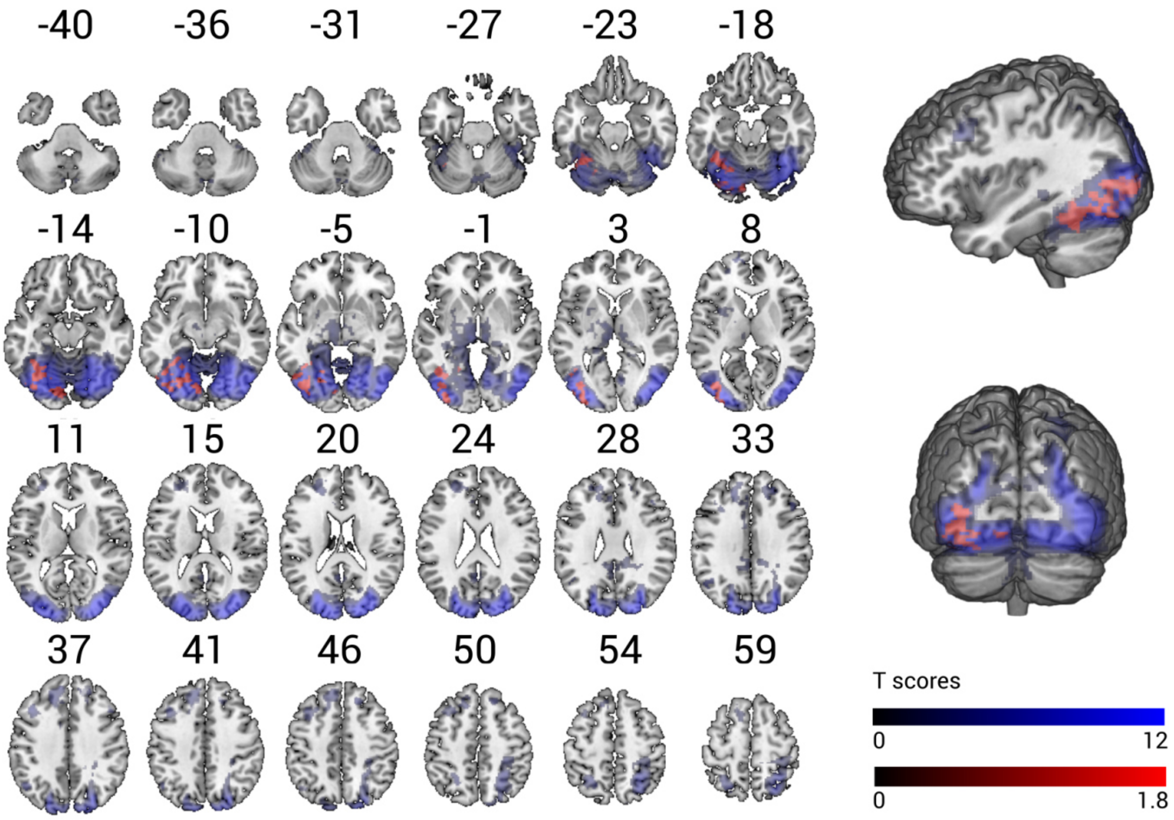

Figure S2. IFC in visual network I in older healthy participants. The clusters significantly related to phasic alerting effects (red) are overlaid on intra-network iFC (blue). The spatial maps are obtained by a combined independent component analysis dual regression approach. Behaviour-iFC associations were tested using a voxel-wise multiple regression, controlling for age, sex, head motion, and education ($p < .05$ FWE corrected at cluster level). The results are presented on a standard anatomical MNI152 template using MRIcroGL (https://www.mccauslandcenter.sc.edu/mricrogl/source); slice numbers in transverse plane are indicated.

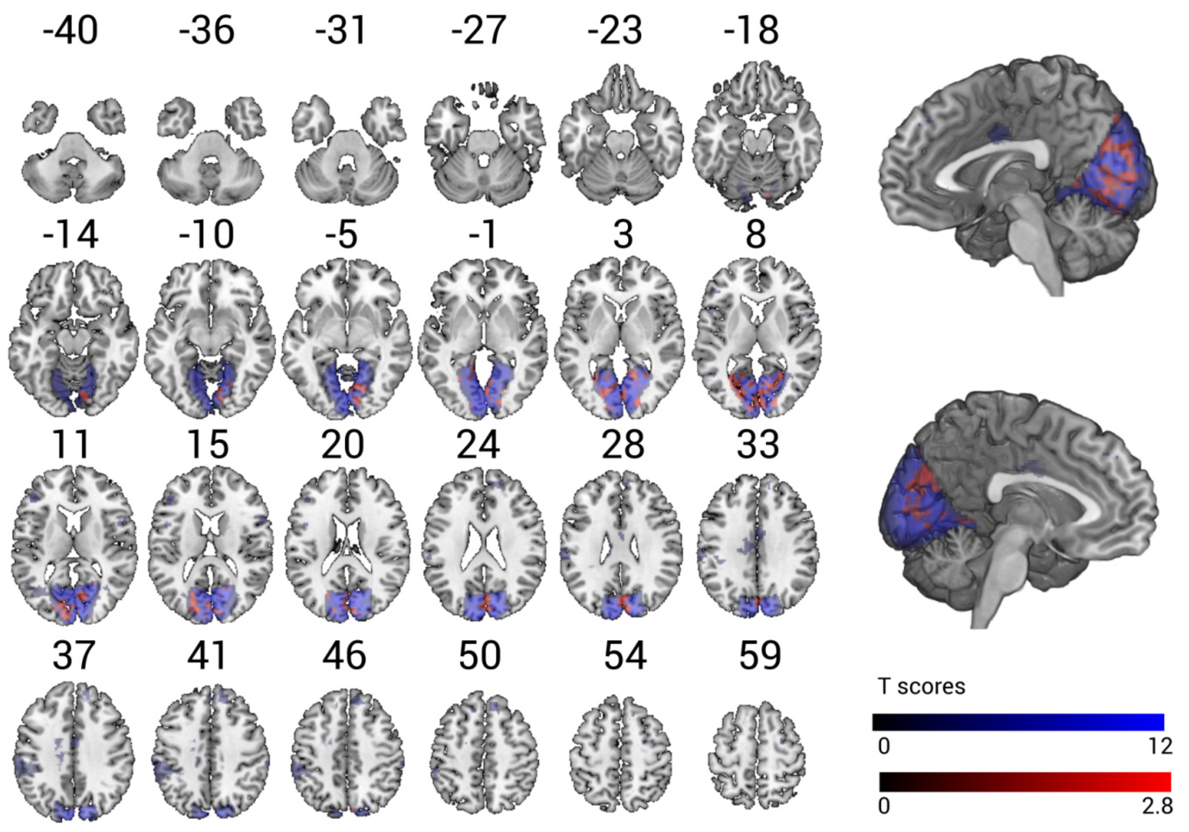

Figure S3. IFC in visual network II in older healthy participants. The clusters significantly related to phasic alerting effects (red) are overlaid on intra-network iFC (blue). The spatial maps are obtained by a combined independent component analysis dual regression approach. Behaviour-iFC associations were tested using a voxel-wise multiple regression, controlling for age, sex, head motion, and education ($p < .05$ FWE corrected at cluster level). The results are presented on a standard anatomical MNI152 template using MRIcroGL (https://www.mccauslandcenter.sc.edu/mricrogl/source); slice numbers in transverse plane are indicated.

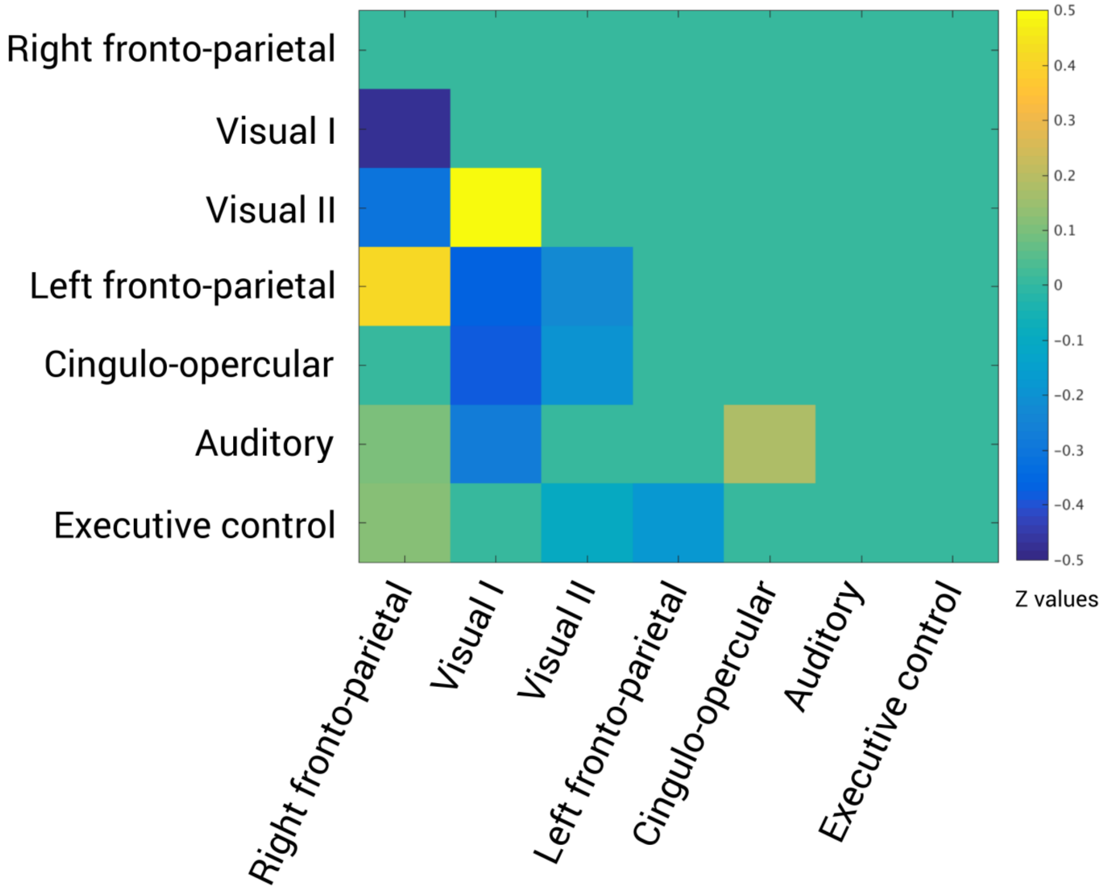

Figure S4. Inter-network connectivity of the cingulo-opercular, right fronto-parietal network, other attention-related, auditory, and visual networks. The figure displays results of one-sample t-tests ($p<.05$, FDR corrected for multiple comparisons) of the correlations between all networks. Positive correlations are highlighted by warm colours; negative correlations are represented by cool colours. The colour bar indicates mean Fisher r-to-z transformed values.

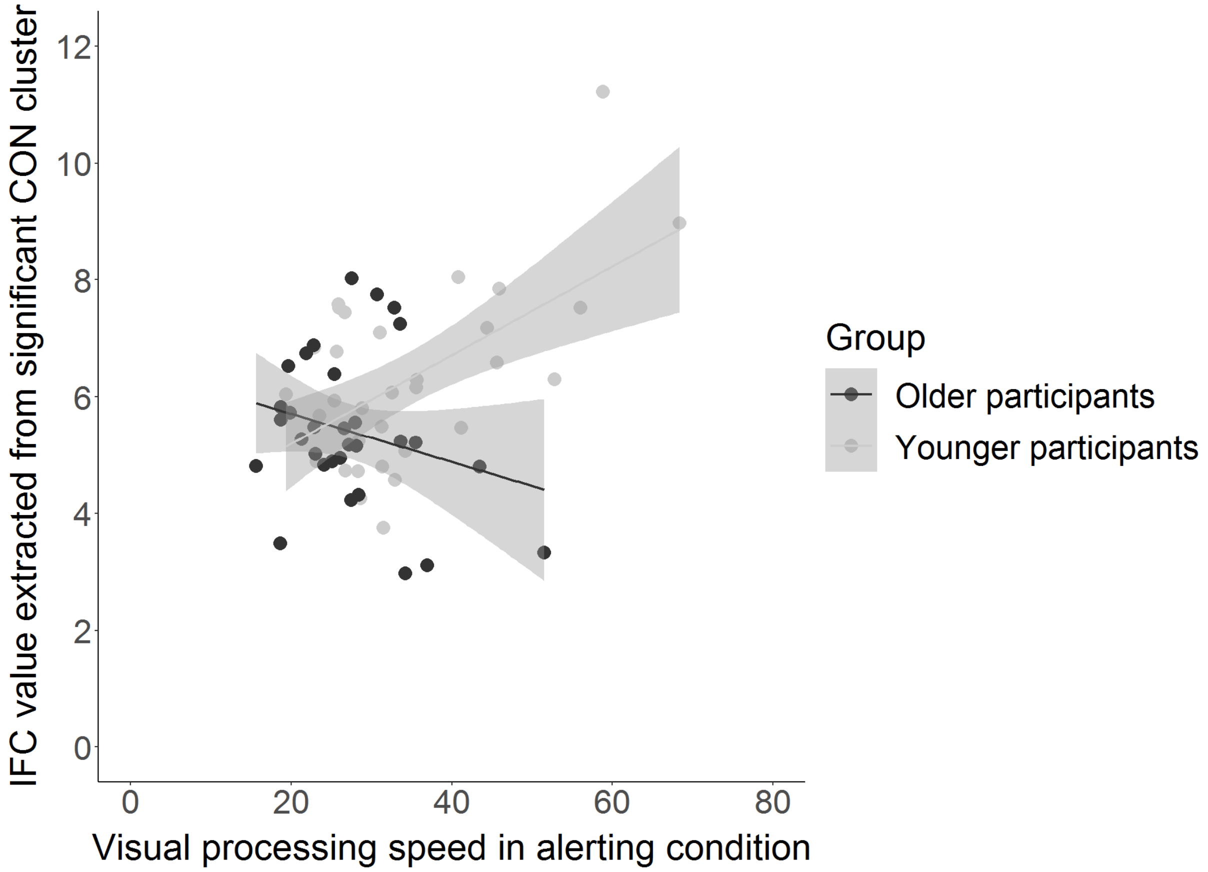

Figure S5. Age group specific behaviour-iFC associations in the cingulo-opercular network. Intrinsic functional connectivity values are extracted from the significant cluster in the cingulo-opercular network (peak MNI coordinates in mm: [14 40 16], cluster size: 1061 voxels, T= 4.20, Z=3.90, $p<.05$ FWE cluster-corrected). The shaded areas around the linear regression lines represent 95% confidence regions.
